# AllergoOncology: ultra-low IgE, a potential novel biomarker in cancer—a Position Paper of the European Academy of Allergy and Clinical Immunology (EAACI)

**DOI:** 10.1186/s13601-020-00335-w

**Published:** 2020-07-17

**Authors:** D. Ferastraoaru, H. J. Bax, C. Bergmann, M. Capron, M. Castells, D. Dombrowicz, E. Fiebiger, H. J. Gould, K. Hartmann, U. Jappe, G. Jordakieva, D. H. Josephs, F. Levi-Schaffer, V. Mahler, A. Poli, D. Rosenstreich, F. Roth-Walter, M. Shamji, E. H. Steveling-Klein, M. C. Turner, E. Untersmayr, S. N. Karagiannis, E. Jensen-Jarolim

**Affiliations:** 1 Department of Internal Medicine/Allergy and Immunology, Albert Einstein College of Medicine, Montefiore Medical Center, Bronx, NY USA; 2 St. John’s Institute of Dermatology, School of Basic & Medical Biosciences, King’s College London, Guy’s Hospital, 9th Floor, Guy’s Tower, London, SE1 9RT UK; 3 School of Cancer and Pharmaceutical Sciences, King’s College London, Guy’s Hospital, London, UK; 4 ENT Research Institute for Clinical Studies, Essen, Germany; 5 LIRIC-Unite Mixte de Recherche 995 INSERM, Universite de Lille 2, CHRU de Lille, Lille, France; 6 Division of Allergy and Clinical Immunology, Department of Medicine, Brigham and Women’s Hospital, Harvard Medical School, Boston, MA USA; 7 Recepteurs Nucleaires, Maladies Cardiovasculaires et Diabete, Univ. Lille, Inserm, CHU Lille, Institut Pasteur de Lille, U1011-EGID, 59000 Lille, France; 8 Division of Gastroenterology, Hepatology and Nutrition Research, Department of Medicine Research, Children’s University Hospital Boston, Boston, MA USA; 9 Randall Centre for Cell and Molecular Biophysics, School of Basic & Medical Biosciences, King’s College London, New Hunt’s House, London, SE1 1UL UK; 10 Medical Research Council & Asthma UK Centre in Allergic Mechanisms of Asthma, London, UK; 11 Department of Dermatology, University of Luebeck, Luebeck, Germany; 12 Interdisciplinary Allergy Outpatient Clinic, Department of Pneumology, University of Luebeck, Luebeck, Germany; 13 Division of Clinical and Molecular Allergology, Research Center Borstel, Leibniz Lung Center, Airway Research Center North (ARCN), German Center for Lung Research (DZL), Borstel, Germany; 14 Department of Physical Medicine, Rehabilitation and Occupational Medicine, Medical University of Vienna, Vienna, Austria; 15 Pharmacology and Experimental Therapeutics Unit, The Institute for Drug Research, School of Pharmacy, Faculty of Medicine, The Hebrew University of Jerusalem, Jerusalem, Israel; 16 Division of Allergology, Paul-Ehrlich-Institut, Federal Institute for Vaccines and Biomedicines, Langen, Germany; 17 Department of Infection and Immunity, Luxembourg Institute of Health, Esch-Sur-Alzette, Luxembourg; 18 The Interuniversity Messerli Research Inst, Univ. of Vet. Medicine Vienna, Med. Univ. Vienna, Univ. Vienna, Vienna, Austria; 19 Immunomodulation and Tolerance Group, Imperial College London, and Allergy and Clinical Immunology, Imperial College London, London, UK; 20 Department of Dermatology, Allergy Division, University Hospital Basel, Basel, Switzerland; 21 Barcelona Institute for Global Health (ISGlobal), Barcelona, Spain; 22 Universitat Pompeu Fabra (UPF), Barcelona, Spain; 23 CIBER Epidemiología y Salud Pública (CIBERESP), Madrid, Spain; 24 McLaughlin Centre for Population Health Risk Assessment, University of Ottawa, Ottawa, Canada; 25 Institute of Pathophysiology and Allergy Research, Medical University Vienna, Vienna, Austria; 26 NIHR Biomedical Research Centre at Guy’s and St. Thomas’ Hospitals and King’s College London, Guy’s Hospital, King’s College London, London, UK

**Keywords:** IgE, Allergy diagnosis, Atopy, Cancer, Malignancy

## Abstract

Elevated serum IgE levels are associated with allergic disorders, parasitosis and specific immunologic abnormalities. In addition, epidemiological and mechanistic evidence indicates an association between IgE-mediated immune surveillance and protection from tumour growth. Intriguingly, recent studies reveal a correlation between IgE deficiency and increased malignancy risk. This is the first review discussing IgE levels and links to pathological conditions, with special focus on the potential clinical significance of ultra-low serum IgE levels and risk of malignancy. In this Position Paper we discuss: (a) the utility of measuring total IgE levels in the management of allergies, parasitosis, and immunodeficiencies, (b) factors that may influence serum IgE levels, (c) IgE as a marker of different disorders, and d) the relationship between ultra-low IgE levels and malignancy susceptibility. While elevated serum IgE is generally associated with allergic/atopic conditions, very low or absent IgE may hamper anti-tumour surveillance, indicating the importance of a balanced IgE-mediated immune function. Ultra-low IgE may prove to be an unexpected biomarker for cancer risk. Nevertheless, given the early stage of investigations conducted mostly in patients with diseases that influence IgE levels, in-depth mechanistic studies and stratification of malignancy risk based on associated demographic, immunological and clinical co-factors are warranted.

## Highlights

1. Elevated IgE is a robust biomarker in atopy, allergy and parasitic infestations, but the implications of ultra-low IgE levels are not understood.
2. Epidemiologic analyses and in vitro and in vivo studies indicate that natural IgE has a surveillance function in cancer, and recombinant anti-cancer IgE is under investigation in a human trial.
3. IgE immunodeficiency correlates with a significantly elevated risk of malignancy development, prompting the need for further research to evaluate ultra-low IgE levels as a new biomarker in oncology.

## Methods

This Position Paper was prepared by the EAACI Task Force (TF) for AllergoOncology, an expert panel of immunologists, allergists, physicians, biochemists and epidemiologists. In the first meeting, topics were identified and prior to the second workshop, a PubMed literature search of specific topics was conducted by individual TF members. The compiled manuscript was extensively revised by the second workshop attendees to obtain consensus on text, tables and figures. Although no evidence-based recommendations were made, due to the limited number of published studies on IgE deficiency, future directions were discussed. The manuscript was recirculated for review to all TF members, compiled and again recirculated for complete consensus. The final manuscript was read and approved by all authors and represents an expert consensus opinion with recommendations summarized in the “Highlights box”.

### Data sources, search strategy and study selection

Published peer reviewed studies in English were identified from PubMed. The following key words were used in the search strategy: (allergy* OR atopy* OR *IgE) AND (tumor/tumour OR cancer OR malignancy) AND (IgE OR IgE deficiency). References published within the 2000-2019 timeframe that had not been otherwise identified in the initial search were added where relevant. We reviewed approximately 250 published studies relevant to this paper.

## Part 1. Intro: a brief history of IgE in atopy/allergy, parasitosis and cancer

Immunoglobulin E (IgE) was isolated in 1968 and recognized as the immunoglobulin isotype involved in allergic reactions [1]. Earlier, two groups worked independently on γE antibody (K. and T. Ishizaka) and IgND (recognized initially as a new myeloma protein by SGO Johansson and H. Bennich), which were found to represent the same IgE molecule [2]. Thousands of publications have since shaped our understanding of the role of IgE not only in type I hypersensitivity reactions, but also in parasitosis and other specific immunologic disorders, and more recently in tumour surveillance (Historic milestones are summarized in Table 1).

**Table 1:** Significant historical events related to IgE

Year	Name/References	Contribution
1869	Bakeley [120]	Invented skin tests for allergy diagnosis
1902	Richet and Portier [121]	Described the term anaphylaxis
1906	Von Pirquet [122]	Used the term “supersensitivity without immunity” to describe symptoms of inhalant allergy and positive skin tests
1913	Clowes [123]	Publication of the first successful trial of ragweed subcutaneous immunotherapy
1919	Ramirez [124]	Recognized the phenomenon of sensitization, when reporting new horse allergy in a patient who received blood from a horse-allergic donor
1921	Prausnitz and Küstner [120]	Described Prausnitz–Küstner (P–K) test, known to sensitize the skin of healthy subjects, through a „transferable sensitization factor“
1930	Coca [125]	Introduced the concept of atopy (hereditary tendency to become allergic) and the term “atopic reagin” as the responsible factor
1960+	Fisherman [126]; Mackay [127]	First epidemiological studies of allergy and cancer risk
1964	Ogilvie [128]	Described reagin-like antibodies in animals immune to helminth parasites
1965, 1967	Johansson et al. [129]	A new myeloma protein named IgND found to inhibit P-K activity
1966	Ishizaka [130]	Working on an antiserum that could deplete the P-K activity, containing a molecule named γE
1967	Wide et al. [131]	RAST (Radio-Allergo Sorbent test) assay development
1968	Bennich et al. [1]	Immunoglobulin E discovery announcement
1971	Ishizaka [132]	Description of IgE-mediated histamine release
1971	Augustin [133]	Published one of the first papers describing IgE levels in cancer and non-cancer patients
1974	Ishizaka and Tomioka [134]	First description of IgE-receptors
1992+	Mills et al. [135]; Kallen et al. [136]; Vesterinen et al. [137]; Turner et al. [138]	Some first large-scale prospective epidemiological studies of allergy and cancer risk
1999	Gould et al. [139]	Reported that IgE antibody-dependent cytotoxicity could be used in suppression of ovarian tumours
1999	Milgrom et al. [140]	First randomized placebo-controlled trial using a recombinant humanized monoclonal antibody against IgE (rhuMAb-E25, later named Omalizumab) as treatment in patients with asthma
2005+	Lindelof et al. [141]; Van Hemelrijck M [142]	First epidemiological studies of pre-diagnostic IgE levels and total cancer risk
2008+	Jensen-Jarolim et al. [143]; Penichet and Jensen-Jarolim [120]; Platzer [26]	Defined the new field of AllergoOncology and the potential mechanisms through which IgE has role in tumour surveillance
2011+	Calboli et al. [22]; Schlehofer et al. [21]; Schwartzbaum et al. [20]	First epidemiological studies of pre-diagnostic IgE levels and specific cancer risk
2016	Simpson et al. [144]	Dupilumab, a fully human monoclonal antibody directed against the interleukin (IL)-4 receptor α subunit, used for treatment in patients with moderate-to-severe atopic dermatitis
2018	Karagiannis SN et al. [145]	Initiation of first phase-1 clinical study (NCT02546921) of IgE antibody MOv18 against an ovarian cancer antigen
2019	Ferastraoaru and Rosenstreich [34]	First longitudinal study of pre-diagnostic IgE deficiency and cancer risk
2020	Spicer JF et al. [80]	Interim data of the first phase-1 clinical study (NCT02546921) of IgE antibody MOv18 against an ovarian cancer antigen presented at the American Association for Cancer Research (AACR) Virtual Annual Meeting

Allergic diseases, which are widespread throughout the world, are driven by a T helper 2 (Th2) immune response to allergens, resulting in production of Th2 cytokines and class-switching to IgE. Th2 responses are actually thought to have originally evolved to control extra‐cellular parasites. Nearly one-third of the global population has some type of helminth infection which triggers an immune response characterized by elevated Th2 cytokines, IgE and eosinophilia [3]. Many questions regarding the immunological similarities between allergic and anti-parasitic immunity have been raised, and molecular similarity between some allergenic proteins and those encoded by parasitic genomes have been reported [4, 5]. It has been hypothesized that, since the rate of parasitic infection is significantly lower in most humans today, the specialized Th2 immune system which primarily evolved to recognize parasite antigens, now reacts to innocuous allergens causing atopic disorders [6–8].

Recently, another new role of IgE in tumour immune surveillance has been suggested by many epidemiological studies and in vitro and in vivo studies in murine models [9–11]. In human populations, early epidemiological studies reported inverse associations between self-reported atopy, anti-histamine use and pancreatic cancer incidence in a population aged > 70 years [12], as well as between physician-diagnosed chronic allergy/atopy and brain tumour incidence [13]. In addition, self-reported asthma and fatal prostate cancer risk [14], as well as self-reported asthma/hay fever and colorectal cancer mortality [15], have been reported to correlate inversely. However, one study of postmenopausal women showed no association between self-reported environmental allergies and incident myeloid or lymphoid malignancies [16]. These findings suggest that the relationship between atopy and malignancy is complex and probably depends on specific tumour types and the individual studied populations.

**Fig. 1:**
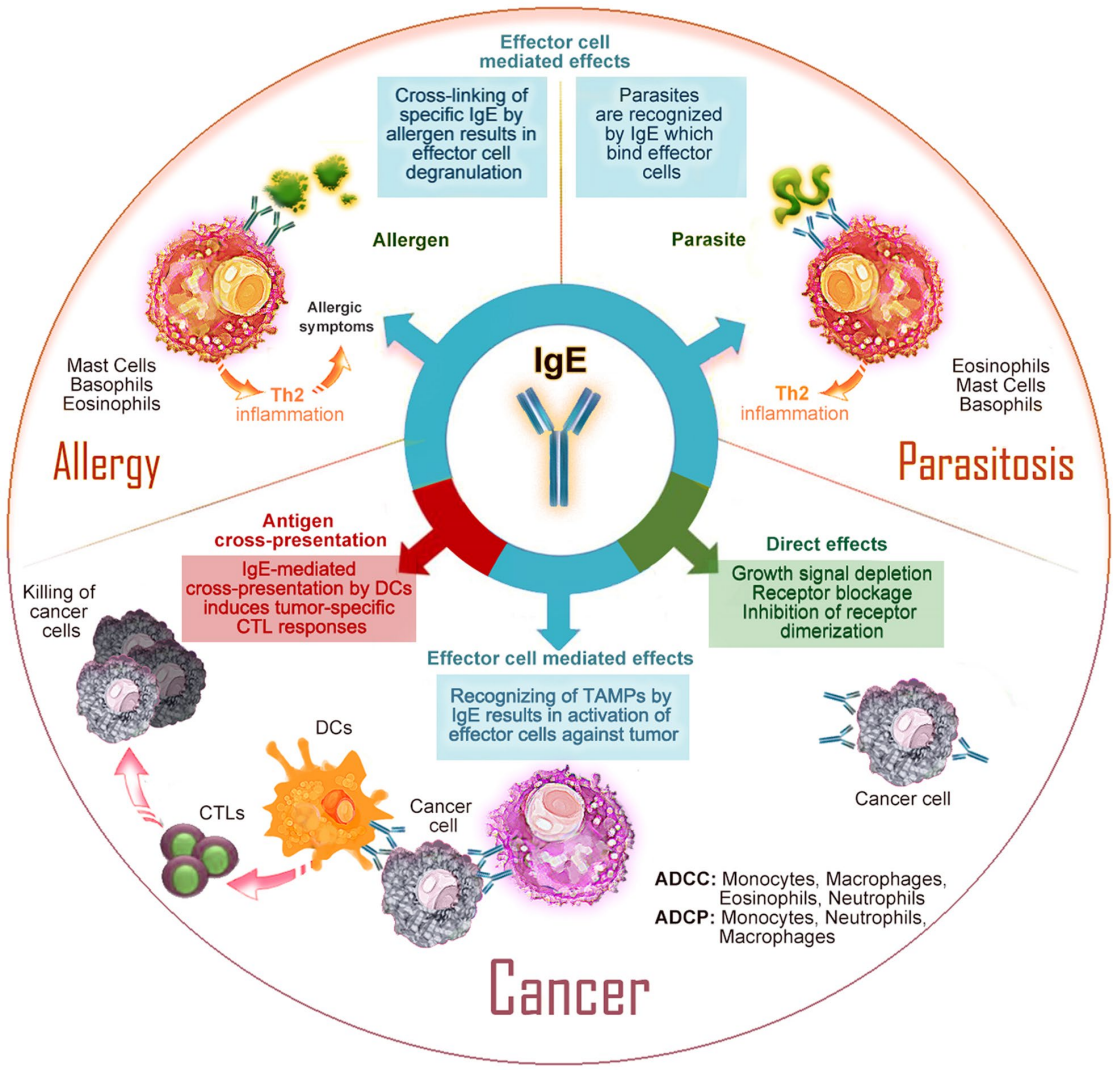
The IgE isotype uniquely encompasses effector function in the pathophysiologies of allergic reactions, parasitosis, and tumour defence

Most of the initial epidemiological studies assessed the association between self-reported allergies and malignancy, which may produce different types of biases. However, once it was recognized that the IgE molecule might be the link between allergies and cancer, different population studies began to examine the association between total or allergen-specific IgE levels and cancer incidence. For example, results from a large Swedish cohort demonstrated an inverse correlation between elevated specific IgE and overall cancer risk, specifically for melanoma, breast and gynaecological cancers [17]. Another prospective study reported that high total serum IgE was associated with a lower risk of developing chronic lymphocytic leukaemia and multiple myeloma [18]. Similarly, higher pre-diagnostic serum IL-4 levels [19], higher total IgE levels [20] and respiratory allergen-specific IgE [21] were associated with a lower risk of developing glioma. However, this was not confirmed in another meta-analysis [22]. Importantly, patients diagnosed with glioma and multiple myeloma with elevated serum IgE experienced longer survival [23, 24]. Despite some mixed results, a 2016 review tabulating the body of epidemiological evidence of the relationship between atopy and cancer risk since 1995, suggested that atopy was associated with a reduced cancer risk [9]. These observations, along with recent murine studies showing that IgE is important in tumour surveillance [11, 25, 26], raise questions on how the incidence and risk of malignancy are affected by ultra-low IgE levels in the clinical practice. To our knowledge, this is the first review that discusses not only consequences of elevated IgE levels, but also the potential clinical significance of ultra-low serum IgE. Figure 1 provides an overview of the diverse role of IgE in atopy, parasitosis and cancer, mechanisms that will be discussed later in this review.

### Overview of medical conditions which are associated with altered serum IgE levels, ranging from ultra-low to elevated serum IgE titres

Elevated IgE (often referred to high and very high serum IgE levels) is commonly considered a serum signature for allergic/atopic conditions [27, 28]. In clinical practice, these patients are referred to Allergy clinics for suspected allergic disorders or other conditions associated with elevated IgE levels. For example, atopic dermatitis and hyper-IgE syndrome are among the disorders with the highest serum IgE levels (often IgE greater than 10,000 kU/L), followed by asthma, parasitosis and allergic rhinitis [29–31].

Total serum IgE of 100–120 kU/L is typically considered the normal upper limit in adults [28], although IgE levels < 100–120 kU/L cannot be used to exclude atopy or allergic disorders. Because of the wide range of IgE levels in non-allergic individuals and in patients with different disorders, no certain cut-offs to define “high IgE” and “very high IgE” states are established. Some studies defined IgE values of 100-1000kU/L as “high IgE level”, and IgE ≥ 1000 kU/L as “very high” [32–34]. Others categorized serum IgE levels of 400–1000 kU/L as “moderately elevated IgE levels”, while IgE > 10,000 kU/L was labelled as “extraordinarily high serum IgE” [29] or “extremely elevated IgE” [35]. We propose a unified classification of different total IgE cut-off levels and their possible associated pathologies (Table 2).

**Table 2:** Definition of different total serum IgE levels cutoffs

IgE deficiency Ultra-low/absent total serum IgE levels (kU/L) IgE < 2.5	Normal total serum IgE levels (kU/L) 2.5 ≥ IgE < 100	High total serum IgE levels (kU/L) 100 ≥ IgE < 1,000	Very high total serum IgE levels (kU/L) 1,000 ≥ IgE < 10,000	Extremely high total serum IgE levels (kU/L) IgE ≥ 10,000
Very low or absent IgE levels may be found in patients with allergic rhinitis-, chronic sinusitis-like symptoms and asthma [32, 36, 37, 58]	Cannot exclude atopic/allergic or parasitic conditions	Biomarker for atopy/allergy [29, 44, 45, 53, 55], parasitosis [71–73]		
Might potentially be used as a marker for higher cancer susceptibility [32–34]	Some patients with normal IgE levels may have higher rates of malignancy [18, 20]	In general, elevated IgE levels are associated with lower rates and risk for malignancy [17, 20, 21, 142]. Very high and extremely high total serum IgE levels are seen in certain malignancies such as lymphoma [146] and IgE myeloma [147]		
May be a sensitive and specific marker for CVID (Common Variable Immunodeficiency) when there is high suspicion for primary humoral deficiency [38]	Cannot exclude immunodeficiency	Elevated IgE levels may raise suspicion for certain immunodeficiencies in appropriate patients (e.g. HyperIgE syndrome (HIES), Wiskott-Aldrich syndrome; immunodysregulation, polyendocrinopathy, enteropathy, X-linked (IPEX); Omenn syndrome; Atypical complete DiGeorge syndrome) [35, 83, 86]		

While research has focused primarily on elevated IgE serum levels, the evidence presented in this Position Paper indicates that investigating the consequences of IgE deficiency (IgE < 2.5 kU/L [36] or IgE < 2 kU/L [37]) may be equally important. Current evidence suggests that IgE deficiency is associated with several types of clinical pathologies, including higher rates of malignancy [32–34, 37]. In selected patients suspected of a primary humoral immunodeficiency, low IgE may be a sensitive and specific marker for the presence of common variable immunodeficiency [38]. Although the pathophysiology of IgE deficiency is not completely understood, it is clear that there is a mechanistic difference between IgE deficiency (ultra-low IgE levels) and atopy (elevated IgE levels) which ultimately may be responsible for the features of these two different conditions. The purpose of this paper is to review for the first time, how different IgE serum levels (from ultra-low to very high) are suggestive of specific pathological conditions, focusing on the less-appreciated anti-tumour role of IgE.

## Part 2: IgE synthesis and regulation

B cells diversify the antibody repertoire by rearrangement, recombination and somatic mutation of immunoglobulin genes. The germline genes are transformed at two stages of B cell development: during V(D)J recombination in B cell precursors, and by somatic hypermutation (SHM) in the Ig variable (V) domains and class switch recombination (CSR) in mature B cells. CSR to IgE requires IL-4 or IL-13 during cognate interactions between CD40-ligand (CD40L) on the Th2 cell and CD40 on B cells, or T cell-independent (TI) stimulation of polyclonal IgE through homologues of CD40L and CD40 in mucosal tissues [39].

There are two types of IgE receptors, high (FcεRI) and low (FcεRII) affinity, that are well described in the literature. In addition, soluble isoforms of IgE Fc receptors (sFcεR) have been described and may form a serum sink for IgE, preventing loading of IgE to surface-expressed FcεRI. In allergic reactions, this may be part of the negative feedback loop that can shut down allergic responses by inhibiting surface binding of IgE and preventing cellular sensitization [40]. On the other hand, the same mechanism could be responsible for preventing IgE-mediated anti-cancer responses and might therefore be considered a feature of tumour evasion. The low affinity receptor for IgE, FcεRII/CD23, which is mainly expressed by B lymphocytes and involved in regulation of IgE production, is expressed on monocytes and follicular dendritic cells that participate in antigen presentation by internalization of IgE-antigen immune complexes. CD23 is expressed in some malignancies (e.g. chronic B cell lymphoma) and serum levels of sCD23 have been described as potential biomarker for cancer progression [41].

## Part 3: Elevated IgE, a biomarker in allergies

### Definition of atopy, allergic sensitization, and allergy

As last published in 2004, “atopy” is defined as “*a personal and/or familial tendency, usually in childhood or adolescence, to become sensitized and produce IgE antibodies in response to ordinary exposures to allergens, usually proteins. As a consequence, these persons can develop typical symptoms of asthma, rhinoconjunctivitis, or eczemas*” [42]. Despite extensive research to find a genetic signature for atopic individuals, no clear genetic fingerprint has yet emerged. An individual with high total or specific serum IgE is considered sensitized, though may not suffer from an allergic disease [43]. The clinical relevance of IgE levels in different type I hypersensitivity disorders is summarized below and in Table 3.

**Table 3:** Medical conditions associated with elevated or very low/absent IgE levels

	Type I hypersensitivity	Parasitosis	Cancer	Other conditions associated with elevated IgE levels
Elevated total and specific IgE levels	*Asthma and allergic rhinitis* In asthma, omalizumab treatment is dosed based on total IgE [56] Total IgE level is useful in diagnosis and monitoring of ABPA flares [44] ssIgE levels depict environmental sensitizations and could guide specific immunotherapy [57] *Food allergy* ssIgE levels to certain foods/molecules predict risk of anaphylaxis [60] *Venom allergy* ssIgE used in venom allergy diagnosis [63] *Drug allergy* ssIgE to penicillin may be useful to diagnose recent anaphylaxis [65] ssIgE to alpha-gal associated with cetuximab anaphylaxis [66]	Ascariasis Schistosomiasis Strongyloidiasis Geohelminthiasis Trichuriasis Enterobiasis IgE levels decrease significantly after effective anti-parasitic therapy [73]	IgE myeloma [147] Hypereosinophilic syndromes [148] Lymphoma [146, 149, 150]	*Atopic dermatitis* May be associated with extremely high IgE levels [29] *Immunodeficiency* [83] HyperIgE syndrome, Wiskott Aldrich syndrome, Immunodysregulation polyendocrinopathy enteropathy X-linked syndrome (IPEX), Omenn syndrome, Atypical complete DiGeorge syndrome *Chronic spontaneous urticaria* Anti-IgE therapies support the role of IgE in pathophysiology [101, 151] *IgE-mediated autoimmunity* (systemic lupus [152], bullous pemphigoid [153], pemphigus vulgaris [154], autoimmune uveitis [155], rheumatoid arthritis [156], multiple sclerosis [157], autoimmune pancreatitis [158]) *IgG4-related disease* [87] *Eosinophilic granulomatosis with polyangiitis (Churg-Strauss)* [88]*, Wegener’s granulomatosis* [159]*, sarcoidosis* [160] *Viral infections* [89, 161]

	Type I hypersensitivity	Parasitosis	Cancer	Other conditions associated with low IgE levels
Very low (< 2.5 kU/mL) or absent total IgE levels	The full clinical significance of very low IgE levels is unknown May cause false negative skin tests and ssIgE results despite clinical suspicion of atopy/allergy The rates of asthma and allergic rhinitis diagnoses are lower compared with non-IgE deficient patients, however, allergy-type symptoms are not absent [32, 37, 58]	It is unknown if humans with IgE deficiency would have higher rates or risk of developing parasitic infections	Higher rates and risk of malignancy compared with non-IgE deficient patients [32–34, 37]	Common Variable Immunodeficiency (CVID) [38] Selective IgE deficiency [162] Hyper-IgM syndrome [163]

*ssIgE* serum specific IgE

### IgE and other markers used in the clinical diagnosis of allergic reactions

Specific environmental, food or drug allergen provocation tests remain the gold standard for the clinical diagnosis of IgE-mediated reactions. However, there are certain conditions under which provocation tests should not be performed, depending on the type of reaction, the results of additional tests and history, and the clinician’s familiarity with this procedure. Therefore, in clinical practice, different tools are used to diagnose IgE-mediated reactions, in addition to detailed history and examination.

Commonly, serum total and allergen-specific IgE (sensitization is defined as serum specific IgE (ssIgE) > 0.35 kU/L) are considered important biomarkers in allergy, atopy and asthma [44–46] (detailed below). Increased mast cell degranulation and clinical symptoms are the result of a number of factors including a high IgE affinity for allergens [47], increased concentration of allergen-specific IgE titers relative to IgE that is not specific for any known allergen, increased serum total IgE, and increased number of epitopes recognized by the IgE repertoire [48].

For some allergens, dominant allergenic proteins have been identified, purified, and incorporated into diagnostic in vitro tests, termed “component-resolved diagnosis” (CRD). Apart from the fact that CRD is often more sensitive than whole allergen extract diagnosis, the presence of specific IgE to certain allergen components is a predictive biomarker for the severity of allergic reactions [49].

Similarly, positive skin tests are considered to be a reliable method for diagnosing inhalant, food, drug or venom allergies [50]. Measuring tryptase levels, during and after an allergic reaction, can differentiate between IgE-mediated hypersensitivity and primary mast cell disorders [51]. Secondary eosinophilia (> 500 cells/µl) may also be found in different atopic/allergic conditions, [52] and periostin might identify asthmatic patients with an IL-13—dependent Th2 phenotype [53]. The basophil activation test, which uses the surface expression of CD63 and/or CD203C following allergen stimulation [54], is emerging as a promising tool to differentiate between true IgE-mediated allergy and allergic sensitization.

### Total and specific IgE measurements as biomarkers in respiratory allergies

Total serum IgE > 100 kU/L was associated with new‐onset asthma in a longitudinal analysis of the European Community Respiratory Health Survey [45], while IgE levels of 200 IU/mL had 93% sensitivity and 91% specificity for asthma diagnosis in another study [55]. This evidence supports the use of anti-IgE as additional therapy in asthma. The dose and frequency of administration of omalizumab, a recombinant humanized IgG1 monoclonal antibody that binds IgE with high affinity, are based on baseline total serum IgE levels (30–1500 IU/ml depending on age), patients’ weight, and sensitization status [56].

Allergic bronchopulmonary aspergillosis (ABPA) is the only allergic disease for which total IgE levels are part of the diagnostic criteria (IgE > 417 kU/L in asthmatics; IgE > 1000 kU/L in cystic fibrosis patients). A 35–50% decrease in total IgE levels in response to treatment suggests remission, while doubling of the baseline IgE levels suggest ABPA relapse [44].

Regarding the utility of performing skin tests and/or measuring specific IgE for diagnosis of environmental allergies, it was shown that, regardless of the allergen tested, every third to fourth patient would have been misdiagnosed as non-sensitized for a particular allergen if diagnosis relied exclusively on serum specific IgE or skin prick testing. These data support the importance of combining skin tests with measurements of ssIgE for diagnostic purposes, and suggested that the two testing methods should be used in a complementary fashion [57]. Nevertheless, other studies showed that levels of ssIgE to dust mites may predict the severity of allergic rhinitis: patients with mild intermittent symptoms had significantly-lower dust mite-specific IgE (median, 6.91kU/L) compared with those with mild persistent symptoms (median, 14.2 kU/L) and those with moderate-to-severe persistent symptoms (median, 30.7 kU/L) [46].

Importantly, patients with IgE deficiency can also suffer from allergic rhinitis, chronic sinusitis-like symptoms, or asthma [32, 36, 37, 58]. It has been speculated that baseline IgE production might have a protective mucosal effect [59], so its absence would contribute to the severity of the above-mentioned conditions. Despite very low or undetectable serum IgE titres, local IgE production could explain tissue-specific allergic responsiveness which should be explored in patients with IgE deficiency who present with symptoms consistent with environmental allergies.

### Specific IgE as a biomarker in food allergies

Considerable efforts have been made to define 95% predictive cut-off specific IgE levels (diagnostic decision points (DDPs)) for egg (7 kU/L), milk (15 kU/L), peanut (14 kU/L), soy (65 kU/L), wheat (80 kU/L), and fish (20 kU/L) [60]. However, anaphylaxis was also reported to occur in patients with low or even undetectable specific IgE. In these cases, CRD is helpful in diagnosing and predicting IgE-mediated food allergies [61, 62]. Additional allergy-enhancing co-factors must be taken into consideration when interpreting the results of specific IgE and CRD in food allergy diagnosis.

### Relevance of total and specific IgE in venom and drug allergies

In patients who have experienced venom anaphylaxis despite having negative skin prick tests, venom-specific IgE is used as an alternative test to support decisions for initiating venom immunotherapy. Interestingly, total IgE levels may predict venom allergy severity: individuals with low total IgE levels (< 50 kU/L) had higher rates of loss of consciousness than those with IgE > 250 kU/L [63]. Using CRD may be helpful to differentiate between honeybee and yellow jacket sensitivity in those with double positivity [63].

Although ssIgE to different drugs can be measured, clear evidence to justify their use in the diagnosis of IgE-mediated drug allergies is lacking, because results do not correlate with the outcomes of drug challenges [64]. However, penicillin-specific IgE may be useful in patients with recent anaphylaxis to penicillin [65]. Sensitization to alpha-Gal is associated with IgE-mediated anaphylaxis to the anti-EGFR therapeutic antibody Cetuximab (used in the treatment of certain solid tumours), due to alpha-Gal moiety on the Fab portion of the heavy chain [66], as well as with allergic reactions occurring more than 2 h after ingestion of mammalian meat in those patients previously sensitized [67]. Evidence that IgE is involved in immediate-type drug allergies is supported by the observation that the off-label use of anti-IgE antibody, omalizumab, effectively prevents such reactions during drug desensitization protocols [68, 69].

## Part 4. Total and specific IgE as biomarkers in parasitic infestations

For > 40 years, a protective role of IgE in helminth infections has been postulated: IgE binding to the parasite attracts IgE-receptor bearing cells to the site, resulting in parasite killing [3, 70] (Fig. 1). Similarly to the atopic/allergic status, the response to parasitic infections is also associated with the induction of strong Th2 immune responses, leading to eosinophilia, elevated Th2 cytokines and increased serum IgE (e.g. in a cohort of patients with *Ascaris* infection, total IgE levels reached 13,000 kU/L) [71, 72]. Moreover, total IgE levels significantly decrease after treatment for *E. histolytica, H. nana or A. lumbricoides* [73].

The role for IgE in fighting parasites was also illustrated by the fact that genetically engineered, IgE-deficient mice infected with *T. spiralis* had significantly increased intestinal and muscular worm burden, compared with infected wild-type mice [74]. However, it is not known if humans with IgE deficiency have higher rates or risk of developing parasitic infections. One retrospective study showed similar rates of positive serologies for *Strongyloides, Toxacara* and *Trichinella* in patients with IgE-deficiency (IgE < 2.5 kU/L), compared with those with IgE ≥ 2.5 kU/L. However, the number of performed serologic tests was small [32]. In support of a positive correlation of serum IgE and parasitic defense, Omalizumab therapy has been associated with a modest increase in helminth infection incidence [75].

## Part 5. IgE and its role in tumour surveillance

### Evidence for a physiological role of IgE in cancer protection and cancer treatment

Host defense against parasites is widely considered to be the most significant beneficial function of IgE. However, a growing body of evidence strongly suggests that IgE plays key roles in tumour immune surveillance. Solid tumours are infiltrated by immune cells that express IgE receptors, resulting in anti-tumor antibody-dependent cell-mediated cytotoxicity (ADCC) and antibody-dependent cell-mediated phagocytosis (ADCP) [10]. Additionally, IgE-mediated cross-presentation of different tumour cell antigens by dendritic cells induces anti-tumour cell cytotoxic T cell activation [26] (Fig. 1).

A higher abundance of IgE-expressing cells was reported in head and neck tumours compared with normal mucosa [76]. Patient-derived pancreatic tumour-specific serum IgE potentiated anti-tumour effector functions in vitro [77], supporting the role of IgE in tumour surveillance. Furthermore, expression of the high-affinity IgE receptor gene *MS4A2* by tumour-infiltrating mast cells in lung cancer was associated with a favourable prognosis and correlated with innate anti-tumour immune responses [78].

In a therapeutic context, the very high affinity of IgE for its cognate Fcε receptors and lack of inhibitory IgE Fc receptors suggests the potential for long-lasting and efficient anti-neoplastic effector cell responses, and this has stimulated interest in the clinical application of anti-tumour IgE in cancer treatment [79]. The first phase I clinical trial of a cancer-associated antigen-specific IgE in patients with advanced solid tumours (NCT02546921) is ongoing and promising interim data from this trial were recently presented. Ultimately, further clinical evaluations should provide important information about the anti-tumour functions of IgE [80].

### Very low total serum IgE levels, a potential novel biomarker in malignancies

One clinical question, arising from the findings that IgE and atopy are protective against malignancy, is whether the opposite is also true, that individuals with very low or undetectable IgE levels have a higher prevalence and risk of developing malignancy. In several epidemiological studies assessing the relationship between IgE levels and cancer, patients with higher IgE levels were found to have a lower risk of chronic lymphocytic leukaemia, multiple myeloma or glioma compared with individuals in the lowest IgE level tertile [18] or with those with IgE < 100 kU/L. [20] Other retrospective studies, focusing specifically on the relationship between ultra-low IgE levels and malignancy, found that IgE-deficient individuals (IgE < 2.5 kU/L) had higher rates and risk of having a diagnosis of any type of malignancy compared with non-IgE deficient individuals (IgE ≥ 2.5 kU/L) [32, 33, 37]. Another prospective, longitudinal study found that IgE deficient patients had, after a median 43.5-month follow up, significantly higher rates and risk of developing new malignancy (17.65%) compared with those with IgE levels 100–1000 kU/L (2.63%) and with those with IgE ≥ 1000 kU/L (0%) [34]. Presently, we do not know if the very low or absent IgE levels are caused by the malignancy but develop before the malignancy becomes clinically apparent, or if IgE deficiency develops for other, unknown reasons, and the low IgE levels cause or are part of an immunomodulatory response associated with increased malignancy susceptibility.

The fact that ultra-low IgE levels may be associated with higher malignancy risk is also supported by different murine studies. For example, in a recent study, IgE-, FcεRI- and IL-4-deficient mice developed skin cancers significantly faster than wild-type mice after skin exposure to a known carcinogen. The same study showed that skin exposure to DNA-damaging stress signals potentiated adaptive immune responses that favoured B cell class switching to IgE and restricted tumour growth [25]. Similarly, mammary carcinomas grew significantly faster in low-IgE ΔM1M2 mice lacking the transmembrane/cytoplasmic domain of the IgE-receptors of B-cells, mimicking a reduced serum IgE level phenotype [81]. In striking contrast, tumour growth was inhibited in high-IgE KN1 mice which have four to sixfold increase in serum IgE levels compared to wild type strains. Moreover, tumour challenge following immunization with irradiated cancer cells caused delayed tumour growth in wild-type but not in IgE-knockout mice. Importantly, mice with higher levels of IgE-secreting B cells were completely protected from tumour growth [81]. In another study, HER-2 overexpressing D2F2E2 cells were grafted into mice with different IgE levels and the immune and survival benefits were compared following treatment with anti-HER-2 antibody trastuzumab [11]. Notably, in this study, the innate immune effects of high-IgE levels in the high-IgE KN1 mice were at least as impressive as the effects of an antigen-specific HER-2 vaccine. The KN1 model may, therefore, recapitulate the epidemiologic observations that total IgE levels in atopic individuals appeared protective against tumour growth.

In summary, these studies point to beneficial immune surveillance roles for IgE in cancer, and to a potential link between absent or very low serum IgE levels and malignancy risk. Although in clinical practice omalizumab is designed to decrease serum IgE levels, there is insufficient evidence to determine whether omalizumab influences development or progression of malignancy in the studied populations [82].

## Part 6: Other conditions affecting IgE levels

### Other medical conditions associated with elevated or ultra-low IgE levels

Several immunodeficiencies are associated with very high and extremely high IgE levels due to regulatory T cell dysfunction, T cell oligoclonality and increased IL-4 production (Table 3) [83]. Some of these rare primary immunodeficiencies caused by STAT3 mutations [31, 84] are characterized by extremely high serum IgE levels (often above 10,000 kU/L) and are associated with a pruritic eczematous skin rash, eosinophilia and impairments of other organ systems including intelligence and motor function. These human hyper-IgE syndromes (HIES) include the autosomal dominant Job´s syndrome and autosomal recessive PGM3 (phosphoglucomutase 3) and SPINK5 (Serine Peptidase Inhibitor Kazal Type 5) syndromes, that can be successfully treated with anti-IgE antibody therapy [85] or stem cell transplantation [86].

Elevated IgE levels may be also found in patients with IgG4-related disease. A total IgE > 480 IU/mL at diagnosis distinguished patients with IgG4-related disease from control subjects with 86% specificity, while IgE > 380 IU/mL identified patients with disease relapse with 88% specificity [87]. Contrastingly, although elevated IgE levels in asthmatic patients with eosinophilia may suggest a diagnosis of EGPA (Eosinophilic granulomatosis with polyangiitis (Churg–Strauss syndrome)), IgE levels alone are not a good predictor of EGPA disease activity [88]. Elevated IgE levels have been reported in patients infected with the human immunodeficiency virus (HIV), specifically in advanced stages, possibly attributed to polyclonal stimulation of B cells [89].

In addition to patients with selective IgE deficiency (IgE < 2.5 kU/mL, but normal IgG, IgA and IgM levels), ultra-low IgE levels may also be found in patients with common variable immunodeficiency (CVID) [38], hyper-IgM syndrome and XLA agammaglobulinemia, due to defects in B cell lineage and IgE-isotype class switching.

### Demographic and lifestyle factors influencing IgE levels

A wide variety of factors affect allergy development and may therefore influence IgE levels (Table 4). In a non-selected Austrian adolescent cohort, male gender was associated with higher sensitization frequency (56.8%) compared to females, while family size and growing up on a farm inversely-correlated with ssIgE levels [90]. After multivariant adjustment in a random sample of Danish children, positive skin prick test, airway hyperresponsiveness, atopic dermatitis, and parental predisposition remained significant predictors of total serum IgE [91]. Smokers and those with occupational dust or gas exposure have higher mean logarithmic IgE values than non‐smokers and unexposed individuals [92]. Similarly, in Korean adults, a larger proportion of individuals with total IgE ≥ 150 kU/L and ssIgE ≥ 0.35 kU/L to *D. farinae* were ex- or current smokers [93] and high-risk drinkers [94]. Among a U.S. population cohort aged ≥ 6 years, median IgE levels were higher: for males (54.8 kU/L) than for females (32.1 kU/L); for non-Hispanic Blacks (71.1kU/L) and Mexican-Americans (64.1 kU/L) than for non-Hispanic whites (33.6kU/L); for persons with < 12th grade education (47.5 kU/L), increased poverty (53.3 kU/L) or higher BMIs (body mass index) (43.2 kU/L) [95]. In the 2005–2006 NHANES (National Health and Nutrition Examination Survey) cohort, there were significantly more Caucasian females with IgE deficiency than non-IgE deficient individuals [33].

**Table 4:** Other factors affecting IgE levels

Demographic factors affecting IgE levels	Medications affecting IgE levels	Other factors affecting IgE levels
Age [164] Childcare [165] Gender [92, 94] Number of siblings [166] Race [95] Socio-economic status [95]	Allergen immunotherapy [167] Corticosteroids [103] Dupilumab [102] Ligelizumab [101] Omalizumab [99] Rituximab [168]	Air pollution [169] Microbiota [110, 112] Smoking [166] Vitamin D level [109]

### Therapeutic manipulations affecting total IgE levels

Total serum IgE may be influenced by medications (Table 4). Omalizumab is targeted therapeutically to reduce free IgE to < 10.4 IU/mL [96]. Assessing changes in total IgE levels pre- and post-omalizumab administration has been challenging because of broad variations of these changes (1.9–51.9%), the assay used, and the molar ratio of omalizumab/total IgE [97]. Currently, there is no accurate technique to measure free IgE levels in omalizumab-treated patients. In clinical practice, an initial increase in total IgE after omalizumab administration (e.g. 431% increase over baseline at 16 weeks in paediatric patients [98]) is observed and can be explained by the increased half-life of IgE-omalizumab immune complexes. Additionally, omalizumab may activate IgE-positive memory B cells and induce IgE production [99]. After an initial increase, IgE levels decrease despite omalizumab continuation (e.g. from 431% increase to 281% decrease at 48 weeks [98], or from 168% increase to 74% decrease [100]), likely because of slow elimination of IgE-expressing lymphoblasts and memory cells due to downregulation of IgE receptors [100]. Presently, no clear consensus has been established among experts on how to utilize total pre- and post-omalizumab IgE levels regarding disease monitoring and response. It is yet to be reported how the new humanized IgG1 monoclonal anti-IgE antibody (ligelizumab) could influence IgE levels, since it binds with higher affinity to the IgE Cε3 domain and is designed to achieve superior IgE suppression [101].

Dupilumab, a fully-humanized monoclonal antibody targeting the interleukin-4 receptor α (IL-4Rα) subunit, which is approved for the treatment of asthma, atopic dermatitis and chronic rhinosinusitis with nasal polyps, decreases serum total IgE levels by a mean of 50% at 16 weeks compared with baseline [102]. The implication of this finding for clinical practice remain to be evaluated.

Corticosteroids are used to control different allergic disorders. Since IgE is a biomarker of “allergies”, one would expect that corticosteroids would decrease IgE levels. However, the decrease in IgE does not occur since corticosteroids polarize the immune system towards a Th2 predominance by suppressing IFN-γ (which normally inhibits Th2 differentiation) and inducing IL-12 production (which normally promotes Th1 cytokines), as well as by increasing IgE production by enhancing the expression of CD40 ligand [103].

Allergen immunotherapy induces an initial increase in serum specific and total IgE, followed by a gradual decrease over months or years of treatment [104].

### Other medical conditions in which IgE might play a pathophysiological role

A higher proportion of patients with chronic urticaria (34%) have total IgE > 175 IU/mL compared with controls [105] and multiple IgE autoantigens were found in patients with chronic spontaneous urticaria (CSU) [106]. Additionally, some patients with refractory CSU respond well to anti-IgE therapies, although the exact mechanism remains unclear [107]. The therapeutic success of anti-IgE treatment in CSU suggests a role for IgE in urticaria pathophysiology.

Different antigenic targets of IgE have been described in several medical conditions, including systemic lupus, bullous pemphigoid, pemphigus vulgaris, autoimmune uveitis, rheumatoid arthritis, multiple sclerosis, autoimmune pancreatitis, which led to the new research field of IgE-mediated autoimmunity [108]. However, IgE is not considered a biomarker in any of these diseases.

Lastly, significant experimental evidence points to the role of vitamin D as a regulator of various immune pathways, and hypovitaminosis D might be associated with elevated total IgE levels and a higher rate of different allergic disorders [109].

### Microbiota influences on IgE levels

Different mechanisms, about how microbiota may influence and regulate the innate and adaptive immune responses with emphasis on the Th2 pathway, have been reviewed in detail in our previous AllergoOncology Position Paper [110]. One study showed that germ-free mice have elevated total IgE levels, resulting in higher susceptibility to anaphylaxis than non-germ free mice, and demonstrating the difficulty of achieving oral tolerance in animals with altered microbiota [111]. In humans, low inferior turbinate bacterial diversity was associated with allergic rhinitis and total serum IgE > 200 IU/mL [112]. Asthmatics with small intestine bacterial overgrowth (SIBO) had higher mean IgE levels (348.4 ± 110 IU/mL) which significantly decreased after SIBO treatment, compared with non-SIBO asthmatics [113]. Presently, our observations predominantly describe correlations rather than causations between microbiota alterations, IgE levels, and allergic reactions. Whether altered microbiomes are a cause or effect of allergies requires further in-depth investigations.

## Conclusion

In clinical practice, attention should be paid not only to patients with high IgE levels, but also to those with very low or absent serum IgE. IgE-deficient individuals have higher rates and risk of malignancy, and attention to this observation will close knowledge gaps with regards to questions such as: (1) Should we check serum total IgE levels in all patients? (2) Which assays should we use to monitor patients with ultra-low IgE levels and what are the ethical implications of not monitoring them? (3) What IgE levels protect from malignancies or, maybe even more importantly, which IgE titres are associated with the risk of developing cancer?

Longer prospective studies are needed to delineate the exact clinical consequence of very low or absent serum IgE levels and to assess if ultra-low IgE will emerge as a clinical biomarker for malignancy susceptibility. While the anti-tumour role of IgE and the potential of IgE-based therapy begins to be recognised, the immunological mechanisms causing IgE deficiency are not yet understood. Dysregulated pathways involved in the IgE class-switching process might not only be responsible for the low serum IgE levels but perhaps also for some impaired anti-tumour activity. Based on the available data, we conclude that low IgE may be a novel, unrecognized biomarker for cancer development. However, substantial effort and interactive research by immunologists, allergists, and oncologists will be necessary to determine the exact role of IgE in malignancy susceptibility and whether there is a specific threshold for serum IgE that determines malignancy susceptibility.

Box 1: Unmet needs to define very low/absent IgE as biomarker in cancer

- The exact IgE level threshold that confers protection versus risk for malignancy occurrence remains to be defined.
- It is not yet clear whether ultra-low/undetectable serum IgE is truly a risk factor for developing malignancy, or if IgE levels are low in response to undetected cancer progression. It is possible that an aberrant and unknown immune pathway causes low IgE levels in response to malignancy.
- There is a need for large prospective studies as well as in vitro experiments and in vivo models to further delineate the role of ultra-low IgE as a putative biomarker for malignancy.

Box 2: Pending questions in the field, future directions

- Is antigen-specific and/or total serum IgE involved in cancer surveillance?
- Is an IgE-related immune score helpful to predict malignancy occurrence? What demographic, clinical and/or immunological factors should this score include?
- New genetic and genomic evaluations are needed to determine if there is a familial risk for IgE deficiency.
- Is the prevalence of IgE deficiency age-dependent?
- Should patients at risk of developing malignancy be treated with specific anti-tumour IgE or total IgE to achieve higher anti-tumour surveillance? Is passive intravenous IgE (IVIGE) or IL-4 a safe option?

Box 3: Recommendations for patients with IgE deficiency

- Due to current minimal information in the field, we cannot specify screening tests or establish recommendations for patients with IgE deficiency. However, with the goal of understanding the role of diminished IgE in the general population, certain clinical findings (e.g. presence of lymphadenopathy, splenomegaly) and laboratory tests (e.g. presence of tumour markers [114], C-reactive protein level [115], DNA methylation test [116], cell frequency detection [117], proteome and miRNome profiling [118], presence of circulating exosomes [119]) may be considered as directions for future studies in these patients.

## Data Availability

Data sharing is not applicable to this article as no datasets were generated or analysed during the current study.
